# Familial Occurrence of Anti‐NF155 Autoimmune Nodopathy in Father and Son: Expanding the Spectrum of IgG4‐Related Nodopathies

**DOI:** 10.1155/crnm/1397400

**Published:** 2026-02-23

**Authors:** Roopa Sharma, Ahmed Sabra, Erin Feinstein, Machteld E. Hillen

**Affiliations:** ^1^ Department of Neurology, Rutgers New Jersey Medical School, Newark, New Jersey, USA, rutgers.edu

## Abstract

We report the first documented familial occurrence of Anti‐Neurofascin‐155 (Anti‐NF155) autoimmune nodopathy in both a father and his son. Anti‐NF155 nodopathy can resemble chronic inflammatory demyelinating polyneuropathy (CIDP) but differs in its poor response to standard CIDP treatments such as intravenous immunoglobulin (IVIG) and steroids, instead requiring B‐cell–depleting therapies. A previously healthy 34‐year‐old male presented with a five‐to‐six week history of subacute sensory ataxia, symmetric proximal and distal weakness, and areflexia, preceded by right‐sided Bell’s palsy. Cerebrospinal fluid (CSF) analysis demonstrated albumin‐cytologic dissociation with a markedly elevated protein level of 485 mg/dL, and nerve conduction studies confirmed a demyelinating polyneuropathy. Despite treatment with IVIG and corticosteroids, he showed minimal improvement, and subsequent detection of Anti‐NF155 antibodies in serum established the diagnosis. He improved significantly with rituximab therapy and was able to walk unassisted at 1‐year follow‐up. Notably, his 13‐year‐old son presented with a similar clinical syndrome at the age of 8, characterized by progressive bilateral weakness, sensory ataxia, tremor, elevated CSF protein of 201 mg/dL, diffuse nerve root enhancement on MRI, and demyelinating polyneuropathy on electrophysiologic studies. He was initially treated as CIDP but failed to respond to IVIG and steroids, later testing positive for Anti‐NF155 antibodies, and has since achieved sustained improvement on rituximab maintenance every six months. This report emphasizes the possibility of genetic predisposition in the pathogenesis of Anti‐NF155 nodopathy and underscores the need for further investigation into familial and environmental factors contributing to disease susceptibility.

## 1. Introduction

Autoimmune nodopathy (AN) is a recently recognized diagnostic category as defined by the 2021 European Academy of Neurology/Peripheral Nerve Society (EAN/PNS) guidelines [1]. These disorders are characterized by autoantibodies directed against nodal and paranodal cell adhesion molecules, such as contactin‐1, neurofascin isoforms (neurofascin‐155 [NF155] and neurofascin‐140/186 [NF140/186]), and contactin‐associated protein (CASPR1) [1]. Patients with AN were initially classified under chronic inflammatory demyelinating polyneuropathy (CIDP) but exhibit distinct clinical features and poor response to first‐line CIDP treatments [1–3]. Because the autoantibodies belong to the immunoglobulin G4 (IgG4) subclass, effective treatment often requires B‐cell–depleting agents [1, 2]. Familial cases of AN have not previously been reported. Here, we describe the first documented occurrence of Anti‐NF155 (Anti‐NF155) AN in both a father and son, underscoring the possible role of genetic predisposition in this rare autoimmune disorder.

## 2. Case Presentation

A 34‐year‐old previously healthy Hispanic male presented with five to six weeks of progressive sensory ataxia, proximal and distal weakness more pronounced in the lower limbs, and areflexia, preceded by right‐sided Bell’s palsy. There was no history of antecedent infection. Cerebrospinal fluid (CSF) analysis revealed albumin‐cytologic dissociation with a white blood cell (WBC) count of 3 and markedly elevated protein of 485 mg/dL. Electromyography (EMG) demonstrated a primary demyelinating sensorimotor polyneuropathy with ongoing axonal loss (Tables 1 and 2). Given the 6‐week history of proximal and distal sensorimotor neuropathy, right Bell’s palsy, areflexia, and demyelinating features on electrodiagnostic testing, a provisional diagnosis of acute‐onset CIDP was made. He was treated with a 5‐day course of intravenous immunoglobulin (IVIG) and discharged on corticosteroids. Given the prominent sensory ataxia and high CSF protein, nodal and paranodal antibody testing were sent. At follow‐up 3 weeks later, he continued to deteriorate, becoming unable to walk unassisted. Serum testing was positive for Anti‐NF155 IgG4 antibodies, and the diagnosis was revised to Anti‐NF155 AN. He was started on rituximab, resulting in progressive improvement; at 1 year, he was able to walk independently.

**TABLE 1:** Nerve conduction studies of upper and lower extremities.

**Motor nerve conduction studies**				
**Nerve**	**Location**	**Onset latency (ms)**	**Amplitude (mV)**	**Conduction velocity (m/s)**

L. Peroneal	Ankle	3.58	**0.8**	
	Fibular head	20.4	**0.5**	**16.81**
R. Peroneal	Ankle	**10.56**	**0.3**	
	Fibular head	18.4	**0.4**	**38.3**
L. Tibial	Ankle	**15.6**	**0.1**	
	Popliteal fossa	**NR**	**NR**	**NR**
R. Tibial	Ankle	**NR**	**NR**	**NR**
	Popliteal fossa	NR	**NR**	
L. Median	Wrist	**7.98**	**1.3**	
	Elbow	12.75	**1.1**	**41.9**
L. Ulnar	Wrist	**10.23**	**0.9**	
	B elbow	15.1	**0.6**	**43.1**

**Sensory nerve conduction studies**				
**Nerve**	**Location**	**Onset latency (ms)**	**Amplitude (μV)**	**Conduction velocity (m/s)**

L. Sural	Calf	2.94	**4.9**	**47.7**
R. Sural	Calf	**NR**	**NR**	**NR**
L. Sup peroneal	Ankle	3.9	**4.5**	**36**
R. Sup peroneal	Ankle	**NR**	**NR**	**NR**
L. Med orthodromic	Digit II	2.17	**1.1**	60

**F wave minimum latencies**				
**Nerves**			**Value (ms)**	

Left peroneal nerve			**NR**	
Right peroneal nerve			**NR**	
Left tibial nerve			**NR**	
Right tibial nerve			**NR**	

*Note:* Abnormal values are bolded. Normal values: peroneal motor onset latency < 6.4 ms, amplitude > 2.5 mV, and conduction velocity > 39 m/s. Tibial motor onset latency < 7 ms, amplitude > 2.5 mV, and conduction velocity > 37 m/s. Median motor onset latency < 4.5 ms, amplitude > 5 mV, and conduction velocity > 49 m/s. Ulnar motor onset latency < 4 ms, amplitude > 5 mV, and conduction velocity > 47 m/s. Superficial peroneal sensory amplitude > 6 μV and conduction velocity > 40 m/s. Sural sensory amplitude > 6 μV and conduction velocity > 39 m/s. Median sensory onset latency < 3.5 ms, amplitude > 6 μV, and conduction velocity > 44 m/s. Ulnar sensory onset latency < 3.6 ms, amplitude > 4 μV, and conduction velocity > 45 m/s.

Abbreviation: NR, nonrecordable.

**TABLE 2:** EMG summary table for upper and lower extremities.

**EMG summary table**							
	**Spontaneous**			**MUAP**			**Recruitment**
**Muscle**	**Fib**	**PSW**	**Other**	**Amp**	**Dur.**	**Poly**	**Pattern**

L. Abductor pollicis brevis	None	None	None	Normal	Normal	None	Reduced
R. Abductor pollicis brevis	None	None	None	Normal	Normal	None	Reduced
L. Tibialis anterior	1+	1+	None	Normal	Normal	None	N
R. Tibialis anterior	None	None	None	Normal	Normal	None	N
L. Gastrocnemius (medial head)	1+	1+	None	Normal	Normal	None	Reduced
R. Gastrocnemius (medial head)	1+	1+	None	Normal	Normal	None	Reduced
L. Vastus lateralis	None	None	None	Normal	Normal	None	Reduced
R. Vastus lateralis	None	None	None	Normal	Normal	None	N

*Note:* Positive sharp waves (PSW) are graded as follows: 1+ indicates occasional discharges with needle motion, 2+ several discharges after needle motion, 3+ frequent discharges without needle motion, and 4+ continuous activity. Fibrillation potentials are similarly graded: 1+ denotes single, discontinuous discharges; 2+ few discharges in multiple areas with discontinuous activity; 3+ continuous activity lasting over 30 s in most sites; and 4+ continuous activity in all sampled sites.

The patient’s 13‐year‐old son experienced a similar illness at age 8, presenting with bilateral weakness, sensory ataxia, and tremor. His CSF protein was 201 mg/dL, MRI revealed diffuse nerve root enhancement, and EMG showed chronic, multifocal, nonlength‐dependent sensory and motor demyelinating neuropathy. He was initially diagnosed with CIDP and treated with IVIG and corticosteroids, with minimal benefit. In 2018, Anti‐NF155 IgG4 antibody testing confirmed the diagnosis of AN, and his treatment was switched to rituximab every six months. His clinical course has since shown significant improvement in strength, gait, and balance, supported by improved electrophysiologic studies.

Both father and son shared clinical features of AN rather than CIDP, including prominent sensory ataxia, significantly elevated CSF protein, and poor response to IVIG.

## 3. Discussion

This familial occurrence of Anti‐NF155 AN raises important considerations regarding genetic and environmental susceptibility. In the father, whole exome sequencing (WES) showed that a heterozygous variant of uncertain significance was identified in the Alanyl‐tRNA synthetase 1 (AARS1) gene, which has been associated with Charcot–Marie–Tooth disease, axonal type 2N [4, 5]. Whether this represents a pathogenic link or an incidental finding remains unclear. Previous studies have demonstrated strong associations between Anti‐NF155 AN and HLA alleles, including HLA‐DRB1 and HLA‐DQB1 in European and Japanese populations [6, 7]. We could not retrieve complete HLA typing data for our patient. The unavailability of complete HLA typing data is a major limitation of this case report, as it may have provided additional insight into the genetic basis of the disease. No such studies have been performed in the Hispanic population.

Our report emphasizes the need for heightened clinical suspicion when patients present with sensory ataxia, very high CSF protein, and poor response to standard CIDP treatments, as these features may indicate AN rather than CIDP. Furthermore, the co‐occurrence in father and son highlights the possibility of hereditary predisposition, warranting additional genetic and immunologic research. A better understanding of these mechanisms will aid in identifying at‐risk individuals and refining therapeutic strategies.

## Funding

No funding was received for this study.

## Ethics Statement

We confirm that we have read the Journal’s position on issues involved in ethical publication and affirm that this report is consistent with those guidelines.

## Consent

The patient has provided written informed consent.

## Conflicts of Interest

The authors declare no conflicts of interest.

## Data Availability

The data that support the findings of this study are available on request from the corresponding author. The data are not publicly available due to privacy or ethical restrictions.

## References

[1] Van den Bergh P. Y. , Van Doorn P. A. , Hadden R. et al., European Academy of Neurology/Peripheral Nerve Society Guideline on Diagnosis and Treatment of Chronic Inflammatory Demyelinating Polyradiculoneuropathy: Report of a Joint Task Force—Second Revision, European Journal of Neurology. (2021) 28, no. 11, 3556–3583, 10.1111/ene.14959. 34327760

[2] Martín-Aguilar L. , Lleixà C. , Pascual-Goñi E. et al., Clinical and Laboratory Features in Anti-NF155 Autoimmune Nodopathy, Neurology Neuroimmunology & Neuroinflammation. (2021) 9, no. 1. 10.1212/NXI.0000000000001098 PMC8564865 34728497

[3] Wang W. , Liu C. , Li W. et al., Clinical and Diagnostic Features of Anti-Neurofascin-155 Antibody-Positive Neuropathy in Han Chinese, Annals of Clinical and Translational Neurology. (2022) 9, no. 5, 695–706, 10.1002/acn3.51550. 35313093 PMC9082385

[4] Setlere S. , Jurcenko M. , Gailite L. , Rots D. , and Kenina V. , Alanyl-tRNA Synthetase 1 Gene Variants in Hereditary Neuropathy: Genotype and Phenotype Overview, Neurology Genetics. (2022) 8, no. 5, 10.1212/nxg.0000000000200019. PMC9450682 36092982

[5] Online Mendelian Inheritance in Man (OMIM) , Entry #601065, Baltimore (MD): Johns Hopkins University, https://omim.org/entry/601065#9.

[6] Martinez-Martinez L. , Lleixà M. C. , Boera-Carnicero G. et al., Anti-NF155 Chronic Inflammatory Demyelinating Polyradiculoneuropathy Strongly Associates to HLA DRB15, Journal of Neuroinflammation. (2017) 14, no. 1, 1–6, 10.1186/s12974-017-0996-1, 2-s2.0-85034235855. 29145880 PMC5691853

[7] Ogata H. , Isobe N. , Zhang X. et al., Unique HLA Haplotype Associations in IgG4 Anti-Neurofascin 155 Antibody-Positive Chronic Inflammatory Demyelinating Polyneuropathy, Journal of Neuroimmunology. (2020) 339, 10.1016/j.jneuroim.2019.577139. 31864140

